# Natural evolution of an eardrum bridge in patients with a traumatic eardrum perforation

**DOI:** 10.1007/s00405-013-2499-8

**Published:** 2013-04-27

**Authors:** Zhengcai Lou

**Affiliations:** Department of Otorhinolaryngology, The Affiliated YiWu Hospital of Wenzhou Medical College, 699 Jiangdong Road, Yiwu, 322000 Zhejiang China

**Keywords:** Natural evolution, Traumatic, Tympanic membrane perforation, Pathology

## Abstract

Although the “eardrum bridge” of traumatic tympanic membrane perforations (TMPs) is very little seen, the underlying natural evolution during the healing process are still unknown.The aim of this retrospective study was to evaluate the natural evolution of the “eardrum bridge” of TMPs. The data for 36 patients with barotrauma-associated traumatic TMPs with an “eardrum bridge” between January 2006 and December 2007 were retrieved. The eardrum bridge was completely liquefied due to infection in one patient. The bridge gradually became necrotic and incorporated into the new eardrum in four patients, and the healed eardrum formed a retraction pocket. In nine patients, epithelial hyperplasia occurred on both sides of the eardrum bridge at the edges, and the bridge became incorporated into the new eardrum, which became very thin over time. However, in 22 patients, the eardrum bridge gradually became necrotic, finally forming a yellow crust-like substance and migrating to the external auditory canal (EAC); it was not incorporated into the new eardrum. The closure of the perforation depended on stratified epithelial migration at the perforation edges near the eardrum bridge, resulting in a normal morphology of the healed eardrum. The present study shows that the eardrum bridge has a different natural evolution during the healing process in patients with a TMP. Most eardrum bridges gradually became necrotic and migrated toward the EAC, and stratified epithelial migration occurred at the perforation edges near the eardrum bridge and closed the perforation. However, a few eardrum bridges gradually became necrotic or developed epithelial hyperplasia, then became incorporated into the new eardrum, resulting in the formation of a retraction pocket and the development of atrophy. Thus, long-term follow-up and histological examination of a larger sample is necessary.

## Introduction

Traumatic tympanic membrane perforations (TMPs) may result from various causes (e.g., slap against the ear, barotrauma, or instrumental injury) [1–3]. Curled edges, or an ‘eardrum bridge,’ are typically seen in some patients with a TMP [3–5]. An eardrum bridge is defined as a few remnants of the eardrum in the area of the perforation that are connected by remnants of the eardrum on both sides of the corresponding perforation edge. The eardrum bridge is similar to a small bridge on both sides of the perforation edge and differs from a curled margin. Curled edges have a free edge on one side, and the other side is connected by remnants to the eardrum. However, an eardrum bridge is substantially comprised remnants of the eardrum.

Many previous studies have focused on the management and natural evolution of these curled edges [5–10]. Some scholars have suggested that curled edges may cause non-centripetal epithelial migration and lead to a prolonged healing time and failure to close, and that ideal management should involve recovery of the original position [5–10]. Other scholars have proposed that the squamous epithelium of the curled edges migrates inward to the tympanum, resulting in the development of middle ear cholesteatoma [8, 11]. A few authors found that the curled edges gradually became necrotic and formed a crust, but that the curled edges did not affect eardrum healing [1, 12]. To our knowledge, however, few experimental or clinical studies have mentioned the natural evolution of an eardrum bridge in cases of TMP. The purpose of this study was to evaluate the evolution of an eardrum bridge in patients with a TMP during the spontaneous healing process via otoendoscopy.

## Materials and methods

### Ethical considerations

The study protocol, including access to and the use of medical records, was approved by the Institutional Ethics Committee of Wenzhou Medical College-Affiliated Yiwu Hospital (Yiwu, Zhejiang, China).

### Case selection

The clinical records of patients with a traumatic TMP, who presented to the Otolaryngology Outpatient Clinic of Wenzhou Medical College-Affiliated Yiwu Hospital between January 2006 and December 2007 were accessed through the Records Department of the Hospital. Cases that met the following inclusion criteria were retrieved for analysis: (1) patients older than 14 years with barotrauma-associated TMP with an eardrum bridge (e.g., a slap or blow to the ear, or a blast injury from firecrackers or fireworks); (2) a fresh perforation within 7 days of the injury; (3) adoption of a conservative management approach; and (4) recorded otoendoscopic video images, including the initial visiting time until closure of the perforation or beyond. An eardrum bridge is defined as remnants of the eardrum in the area of the perforation that are connected by remnants of the eardrum on both sides of the corresponding perforation edge. Cases with inadequate documentation of otologic examination findings or early patch or surgical intervention after ear injury were excluded. The following criteria were used to assess the relative size of the TMP: small, <25 % of the TM area; medium, 25–50 % of the TM area; and large, ≥50 % of the TM area.

### Data analyses

All clinical records and otoendoscopic videos of the patients were made available by the Records Department of the Hospital. All recorded video images were imported into a computer for storage. Morphological changes in the eardrum bridge, the size of the perforation, and ultimate healing outcomes were analysed by an independent, blinded reviewer using ImageJ software (AutoCAD R14).

## Results

### Demographic characteristics and healing outcome

Of the 427 patients with a TMP who were diagnosed between January 2006 and December 2007, only 36 (8.4 %) had an eardrum bridge. Thus, 36 outpatients with a traumatic TMP with an eardrum bridge met the inclusion criteria and were analysed. The mean age of these 36 patients was 39 years (range 24–52 years); 32 were males and 4 were females. Thirty-four patients had injuries that resulted from a slap or blow to the ear, and two patients had a blast injury from small firecrackers. No patients had pre-existing tympanosclerosis. Small perforations were observed in 33 (92 %) patients, and medium perforations were present in 8 % of the patients. No large perforations were found. A total of 83 % of the perforations were located in the anterior–superior quadrant, and 17 % were located in the anterior–inferior quadrant.

Among the 36 patients, the follow-up time ranged from 6 months to 3 years with a mean time of 9.1 months. The healing rate was 100 % (33/33) for small perforations, and the closure time ranged from 12 to 38 days with a mean time of 21.2 days. Two patients with medium perforations had complete closure; the closure times were 28 and 31 days, respectively. One patient failed to have closure because of infection.

### Morphological changes in the eardrum bridges

**Fig. 1:**
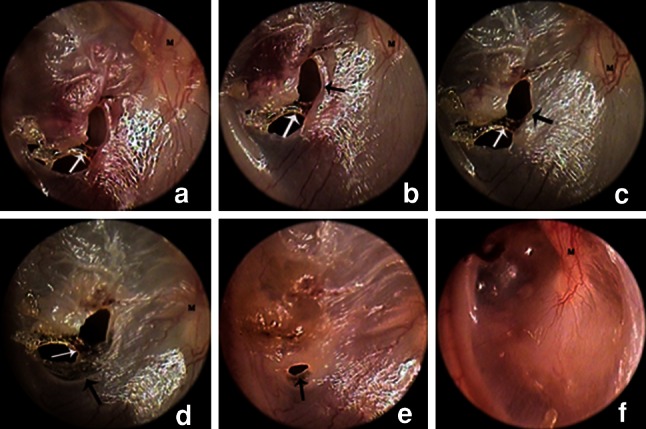
Otoendoscopic images of representative eardrum bridge necrosis and incorporation into new eardrum during the process of spontaneous healing: day 1 (**a**), day 3 (**b**), day 6 (**c**), day 9 (**d**), day 12 (**e**), and day 23 (**f**) (*black arrows* proliferating epithelium, *white arrows* eardrum bridge)

In 4 of the 36 patients, the eardrum bridge gradually became necrotic and formed a black crust. Within 7 days after the injury, thin, transparent proliferation of the epithelium was seen on both sides of the perforation edges. The epithelial proliferation moved toward the centre of the perforation and became increasingly notable over time, completely closing the perforation. However, the necrotic eardrum bridge did not detach from the surface of the eardrum; instead, it became incorporated into the new eardrum. The eardrum bridge appeared to be replaced by the newly formed eardrum. The surface of the healing eardrum was rough in the initial stage. Soon afterwards, its surface became glossy and formed retraction pockets in contrast to the uninjured eardrum (Fig. 1).

**Fig. 2:**
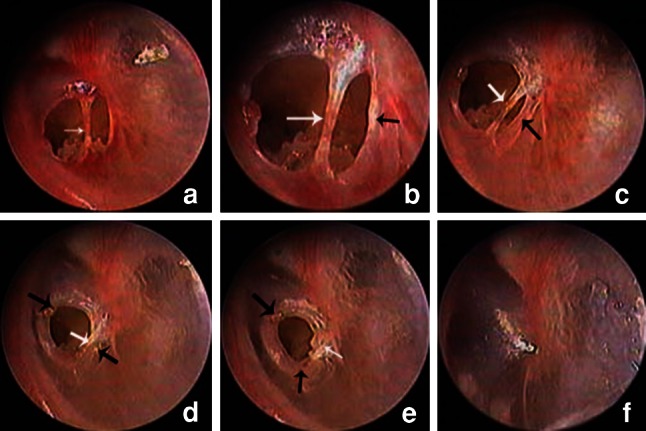
Otoendoscopic images of a representative stratified migration of epithelium during the process of spontaneous healing: day 2 (**a**), day 4 (**b**), day 9 (**c**), day 16 (**d**), day 29 (**e**), and day 49 (**f**) (*black arrows* proliferating epithelium, *white arrows* eardrum bridge)

The eardrum bridges of 23 patients gradually formed a grey crust. The necrotic eardrum bridge completely liquefied and disappeared due to infection in one patient, and the perforation failed to achieve complete closure. In the remaining 22 patients, subsequent epithelial hyperplasia occurred at the perforation margin over time, and stratified migration of the outer squamous epithelium from the eardrum was seen at the perforation margin near the eardrum bridge. The stratified epithelium beneath the eardrum bridge moved toward the centre of the perforation. The stratified migratory epithelium became increasingly notable and eventually closed the perforation completely. The formed crust of the eardrum bridge migrated toward the annulus and EAC. The morphology of the healed eardrum was normal (Fig. 2).

**Fig. 3:**
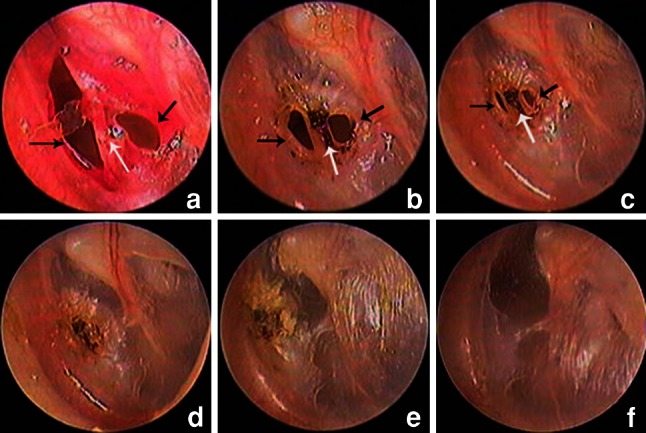
Otoendoscopic images of representative epithelium proliferation and migration of eardrum bridge during the process of spontaneous healing: day 1 (**a**), day 6 (**b**), day 10 (**c**), day 20 (**d**), month 3 (**e**), and year 3 (**f**) (*black arrows* proliferating epithelium, *white arrows* eardrum bridge)

As with the other perforated edges, epithelial hyperplasia occurred on both sides of the eardrum bridge edges in nine patients. As a result of the migration of proliferating epithelium toward the centre of the perforation, the eardrum bridge gradually broadened and the perforation closed completely. The eardrum bridge also became incorporated into the new eardrum; however, the healed eardrum became very thin over time (Fig. 3).

## Discussion

Our study demonstrates that a sudden increase in air pressure within the EAC can cause the formation of an eardrum bridge. We found that eardrum bridges emerged mainly in small and medium perforations, whereas this phenomenon was not seen in some large perforations. It is possible that eardrum bridges in large perforations are divided into several small perforations. Although the incidence of eardrum bridges was low, as a specific pathological phenomenon, its natural evolution is worthy of discussion. This present study shows that eardrum bridges have a different natural evolution during the healing of TMPs.

In most cases, although the eardrum bridge gradually became necrotic, formed a yellow crust-like substance, and migrated to the EAC, the eardrum bridge did not become incorporated into the new eardrum. However, healing of the perforation depended on stratified epithelial migration at the perforation edges near the eardrum bridge, which completely closed the perforation, and the morphology of the healed eardrum was normal. A previous study confirmed stratified epithelial migration in experimental TMP [13, 14] and in human TMP without an eardrum bridge [15].

In this study, a few eardrum bridges were incorporated into the new eardrum. When the width was narrower, the eardrum bridge gradually became necrotic due to the lack of blood supply and was incorporated into the new eardrum. The behaviour of the eardrum bridge seemed to result in retraction of the healed eardrum over time. How the eardrum bridge is incorporated into the new eardrum is not well understood. However, if the width of the eardrum bridge was greater, similar to the other perforation edges, epithelial hyperplasia also occurred on both sides of the eardrum bridge edges, and the width of the eardrum bridge gradually increased and completely closed the perforation. It is possible that the eardrum bridge obtained a sufficient blood supply that resulted in epithelial proliferation on both sides of the eardrum bridge edges, thereby facilitating eardrum healing and incorporation into the new eardrum. Unfortunately, the healed eardrum atrophied and became thinner. It is possible that the absorption of the eardrum bridge inhibited fibrous layer synthesis during the eardrum remodelling phase, but evaluation of the exact mechanism requires further study. In addition, the eardrum bridge became incorporated into the new eardrum and led to retraction pocket formation or atrophy. Under these circumstances, whether the eardrum bridge resulted in the development of middle ear cholesteatoma is unclear due to a lack of imaging and histologic evidence. However, the pathological behaviour alerted us to the need for further observation and study. A previous study indicated that the development of middle ear cholesteatoma usually occurs 12 months or more after post-traumatic TMP [11].

We acknowledge that this study has a number of limitations. First, the number of patients was small. Second, we did not assess the histological changes in the eardrum bridge after it became incorporated into the new eardrum, due in part to a lack of continuous histological specimens. In addition, we did not perform an objective analysis regarding whether the eardrum bridge caused the development of middle ear cholesteatoma because of the relatively short follow-up time and the lack of imaging data. Thus, further clinical and experimental research is necessary.

## Conclusions

The eardrum bridge is a phenomenon sometimes seen in human traumatic TMP. The eardrum bridge gradually becomes necrotic or displays epithelial proliferation; it then becomes incorporated into the new eardrum, resulting in retraction pocket formation or eardrum atrophy. Under these circumstances, whether the eardrum bridge results in the development of middle ear cholesteatoma is unclear. However, in most of the cases in the present study, the eardrum bridge gradually became necrotic and migrated toward the EAC, and complete closure of the perforation depended on stratified epithelial migration at the perforation edges near the eardrum bridge. The morphology of the healed eardrum was normal.
